# Crosstalk between iron and flavins in the opportunistic fungal pathogen *Candida albicans*

**DOI:** 10.1016/j.jbc.2025.110396

**Published:** 2025-06-19

**Authors:** Marika S. David, Zhengkai Zhu, Maranda R. McDonald, Mohsen Badiee, I. Phillip Mortimer, Anthony K.L. Leung, Valeria C. Culotta

**Affiliations:** 1 The Department of Biochemistry and Molecular Biology, Johns Hopkins University Bloomberg School of Public Health, Baltimore, Maryland, USA; 2 Department of Chemistry, Johns Hopkins University, Baltimore, Maryland, USA

**Keywords:** iron, metal, riboflavin, Sef1, yeast

## Abstract

As part of the innate immune response, the host withholds metal micronutrients such as iron (Fe) from invading pathogens. To survive such Fe-limitation, the opportunistic fungal pathogen *Candida albicans* has evolved elaborate responses to Fe-starvation stress. One such adaptation involves the secretion of flavins, yellow isoalloxazine compounds that serve important redox roles in biology. Why the organism secretes flavins during Fe-starvation is not known. Moreover, the exact flavin secreted by the fungus or the effects of Fe-starvation on intracellular flavin pools have not been documented. Major cellular flavins include riboflavin (vitamin B2) and the downstream metabolites and enzyme cofactors FAD and FMN. Of these, our HPLC and mass spectrometry analyses identify riboflavin as the sole flavin secreted by Fe-starved *C. albicans.* Fe also regulates intracellular pools of flavins. While Fe-replete cells have abundant FMN and FAD with only trace riboflavin, Fe-starvation induces a spike in intracellular riboflavin, whereas FMN and FAD are unaffected. These shifts in riboflavin are dependent on the Fe-sensing transcription factor Sef1. During Fe-starvation, Sef1 induces genes for riboflavin biosynthesis but not for the conversion of riboflavin to FMN and FAD. Sef1 is also needed to activate riboflavin export. We provide evidence for the first time that extracellular riboflavin can enhance fungal uptake of Fe. Specifically, riboflavin increased *C. albicans* acquisition of Fe from animal serum, presumably through its redox activity on extracellular Fe. Our observed role of riboflavin on Fe uptake may promote *C. albicans* fitness in the Fe-limiting environment of the host.

Transition metals such as iron (Fe) are essential micronutrients across the tree of life, functioning as cofactors for large families of metalloenzymes. Yet these same metals are potentially toxic, and organisms have therefore developed strategies to balance metal acquisition and detoxification. During infection, hosts exploit microbial dependence on metals such as Fe to combat pathogenesis through processes collectively known as nutritional immunity (1). One infectious microbe subjected to nutritional immunity for Fe is *Candida albicans*, the most prevalent human fungal pathogen. As an opportunistic pathogen, *C. albicans* can exist as a commensal in the mucosa of the digestive tract (2) and can then become infectious and invasive particularly in immunocompromised hosts, with mortality rates up to 30% (3). When *C. albicans* transitions from commensal to infectious, it faces fluctuations in Fe bioavailability that range from abundant Fe in the gut to very low Fe levels in the bloodstream (2, 4, 5). In target organs of infection such as the kidney, Fe-exclusion zones develop at sites of fungal lesions, creating a profound Fe-starvation state for the microbe (6).

In order to balance variable Fe availability inside the host, *C. albicans* has three different pathways of Fe uptake, including a reductive pathway, and receptors for heme/hemoglobin and for Fe^3+^ binding siderophores (7). Extracellular Fe is typically in the oxidized ferric Fe^3+^ state, and in the reductive pathway, the metal is reduced to the Fe^2+^ ferrous state prior to cell uptake, an activity accomplished by metalloreductase enzymes or other cellular reductants (7). In *C. albicans*, the reductive pathway not only works on ionic Fe^3+^ but also on Fe^3+^ bound to macromolecules such as serum transferrin and ferritin (7, 8, 9). All three pathways for Fe uptake in *C. albicans* are regulated at the transcriptional level by Fe and three trans-regulators: Sfu1, Hap43, and Sef1. In the Fe-rich environment of the digestive system, Sfu1 represses Fe uptake, and Hap43 upregulates pathways for Fe storage and utilization (2, 7, 10, 11). When *C. albicans* transitions to Fe-limited environments, Sef1 upregulates the aforementioned Fe uptake pathways, and Fe utilization and storage are repressed (7, 10, 12, 13, 14). Curiously, *C. albicans* Sef1 also strongly upregulates flavin secretion when cells are starved for Fe (15, 16, 17).

Flavins are a class of isoalloxazine ring-containing molecules that function in cellular redox chemistry, an example being riboflavin or vitamin B2 (17, 18). Riboflavin is an essential nutrient synthesized by plants, bacteria, and fungi and serves as a precursor to flavin mononucleotide (FMN) and flavin adenine dinucleotide (FAD) cofactors for redox-active enzymes. With all flavins, the isoalloxazine ring strongly absorbs at ≈373 and 445 nm, producing a bright yellow color (17, 18). A number of studies have noted this trademark yellow color of Fe-starved *C. albicans* cultures, and *C. albicans* has been classified as a “flavogenic” yeast, capable of secreting micromolar levels of flavins under Fe starvation (16, 17, 19). However, the exact flavin(s) secreted by *C. albicans* during Fe-starvation has not been well-characterized. Another flavogenic yeast, *Candida famata* can secrete both FMN and riboflavin (20), and it was not clear if the same was true for *C. albicans*. Furthermore, the impact of Fe-starvation on intracellular pools of flavins has not been investigated. Does Fe only regulate flavin secretion or also shift intracellular pools of riboflavin, FMN, and FAD?

It is important to note that the secretion of flavins with Fe-starvation is not restricted to fungi. Flavin secretion is also known to be inversely regulated by Fe in certain bacteria and plants (21, 22, 23), and the exact flavin secreted ranges from riboflavin, FAD, and FMN in nitrogen-fixing and methanotrophic bacteria to riboflavin 3′ sulfate and riboflavin 5′ sulfate in sugar beet roots (24, 25, 26, 27). Why such diverse flavins would be secreted during Fe starvation is largely unknown, but several theories have been put forth, including promoting Fe uptake through flavin reduction of extracellular Fe^3+^ (21). Data supporting this model is currently lacking.

Here we investigate in detail the connection between Fe and flavins in *C. albicans.* We identify riboflavin as the sole flavin secreted by *C. albicans* during Fe starvation and describe how the Sef1 transcription factor controls both intracellular and extracellular pools of riboflavin, with minimal effects on FAD and FMN enzymatic cofactors. Moreover, we provide evidence for a role for riboflavin in promoting fungal uptake of Fe from serum. These findings underscore the importance of fungal flavins as promising targets for antifungal therapy.

## Results and discussion

### Riboflavin is the sole flavin secreted by *C. albicans*

We aimed to understand the composition of flavins secreted by *C. albicans* during Fe-starvation. In past studies, riboflavin was the suspected molecule secreted by *C. albicans* based on spectroscopic properties of unfractionated growth media, *i.e.*, absorbance at ≈450 nm and/or fluorescence at ≈520 nm (15, 28). However, these same properties are shared among widespread flavins with isoalloxazine rings (18, 26), and flavin identification requires chromatographic resolution and biophysical characterization (29). Here we use high-performance liquid chromatography (HPLC) and mass spectroscopy to identify the flavin(s) secreted by *C. albicans.*

**Figure 1:**
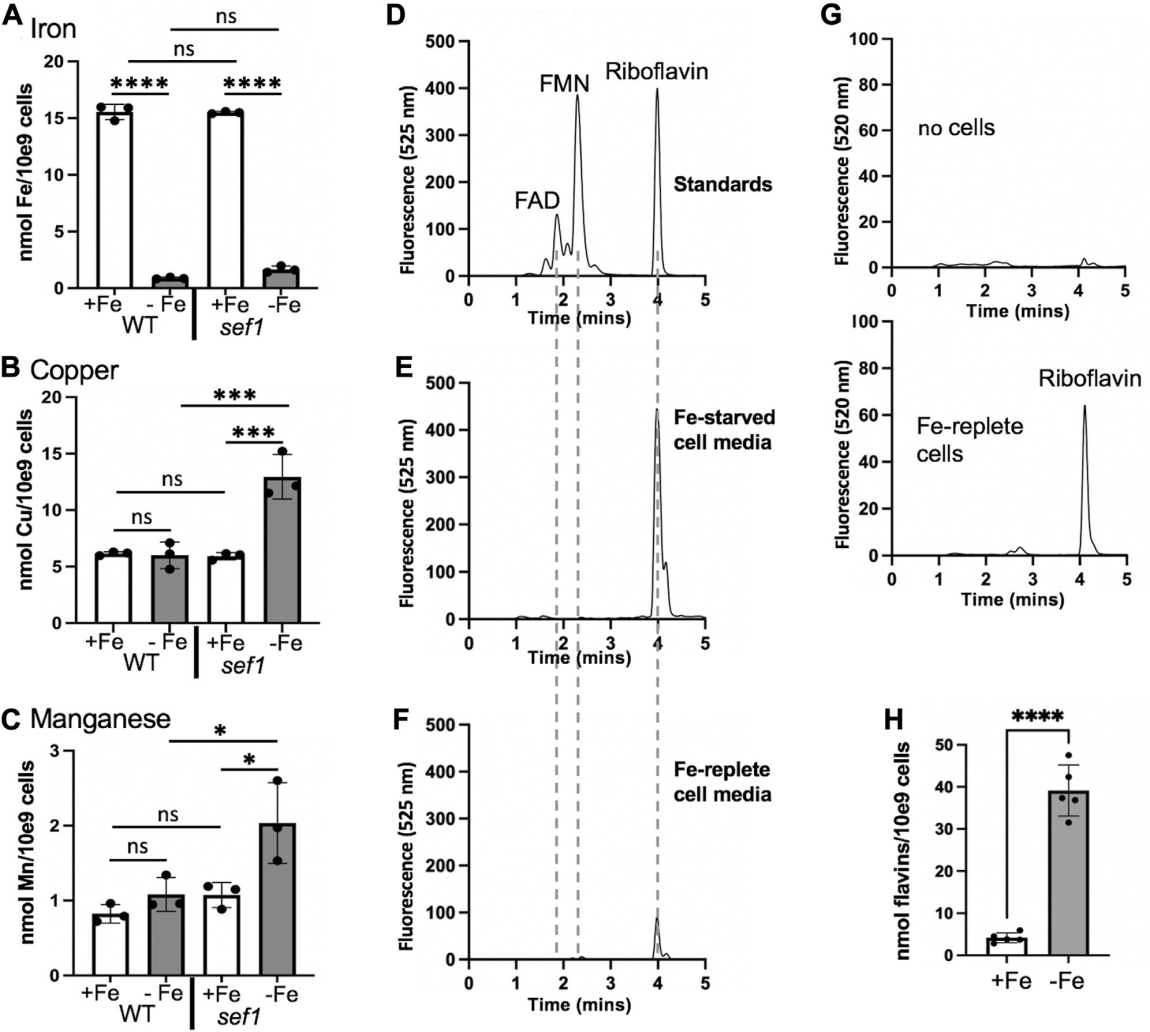
**Riboflavin as the sole flavin secreted by Fe-starved *C. albicans*.** WT strain SC5314 (*A*–*C*, *E*–*H*) or the isogenic *sef1Δ/Δ* mutant (*A*–*C*) was grown under Fe-replete or Fe-starvation conditions and either analyzed for total accumulation of metals (*A*–*C*) or for flavin secretion (*E*–*H*). In part *A*–*C*, whole cell accumulation of Fe or of Mn and Cu was carried out by the BPS or AAS assays, respectively, as described in Experimental Procedures. *D*–*F*, HPLC chromatograms of flavin fluorescence at 525 nm were obtained as described in Experimental Procedures from (*D*) a mixture of 2.0 nmols FAD, FMN and riboflavin standards, or spent media from Fe-starved (*E*) or Fe-replete (*F*) WT cells. *Dotted lines* indicate individual times of elution for FAD, FMN and riboflavin based on standards run in parallel. Note: The decreased intensity of the FAD standard reflects it lower fluorescence constant at 525 nm (see main text for details). *G*, HPLC chromatograph of media alone (“no cells”) *versus* an equal amount of spent media from Fe-replete cultures shows the dependence on cells for the riboflavin fluorescence detected. *H*, The area under the curve (AUC) for the riboflavin peak was used to quantify riboflavin secreted from Fe replete (+Fe) *versus* Fe-starved (−Fe) cells as described in Experimental Procedures. For all bar graphs, each value represents an independent fungal culture. Bars are mean and standard deviation, and significance determined by one way ANOVA with *post hoc* Tukey test (*A*–*C*) or unpaired *t* test (*H*); ∗∗∗∗*p* < 0.0001; ∗∗∗*p* = 0.003; ∗*p* = 0.021 (part *C sef1* +Fe *versus* -Fe) and 0.022 (part *C* WT -Fe vs *sef1* -Fe).

To induce fungal flavin secretion through Fe-starvation, we used a synthetic medium depleted of Fe (14, 30). In this manner, we could obviate the need for Fe-chelators that can react with other metals such as Cu^2+^. As shown in Figure 1*A*, WT cells grown in this Fe-depleted media accumulated ≈15-fold lower levels of Fe compared to the same cells grown under Fe-replete conditions (5 μM FeCl_3_). Importantly, there was no change in intracellular levels of Cu or Mn with this Fe-depleted media (Fig. 1, *B* and *C*). The extracellular media from cells grown under these Fe-starvation conditions or corresponding Fe-replete controls were subjected to HPLC, as was previously done for individual flavin analyses (29). As seen in Figure 1*D*, this method effectively resolves equimolar amounts of FAD, FMN, and riboflavin standards detected by fluorescence at 525 nm. The smaller peak of FAD is consistent with its lower fluorescence constant compared to FMN and riboflavin (31). In the analysis of the spent media from Fe-starved cells, we observed only one peak, which aligns with that of the riboflavin standard (Fig. 1*E*). ESI-mass spectrometry analysis of this fraction shows the characteristic 375 m/z peak of riboflavin, and 255/256 m/z peaks consistent with the lumiflavin hydrolysis product of riboflavin, both of which are absent in the blank control (Fig. S1). Hence, riboflavin is the sole flavin secreted by Fe-starved *C. albicans*, and its production increases up to 24 h of culture (Fig. S2*A*). These findings are in stark contrast to the fungus *C. famata* and nitrogen-fixing and methanotrophic bacteria that secrete FMN and/or FAD as well as riboflavin (20, 24, 25). Additionally, *C. albicans* does not secrete sulfated versions of riboflavin, as has been described for some Fe-starved plants (26, 27).

A low but consistent amount of riboflavin was also detected in the media from Fe-replete cells, shown in Figure 1*F*. The synthetic media used in these studies contains 0.2 mg/L riboflavin; however, this level is poorly detected in our HPLC assay as seen in a direct comparison of media-alone (no cells) *versus* media from Fe-replete cultures (Fig. 1*G*). We conclude that low levels of riboflavin are indeed secreted from Fe-replete cells. Quantification of total riboflavin secreted as a function of cell number in Figure 1*H* shows a greater than 9-fold increase in secreted riboflavin under Fe-starvation compared to Fe-replete conditions.

### The effects of Fe starvation on intracellular pools of flavins

**Figure 2:**
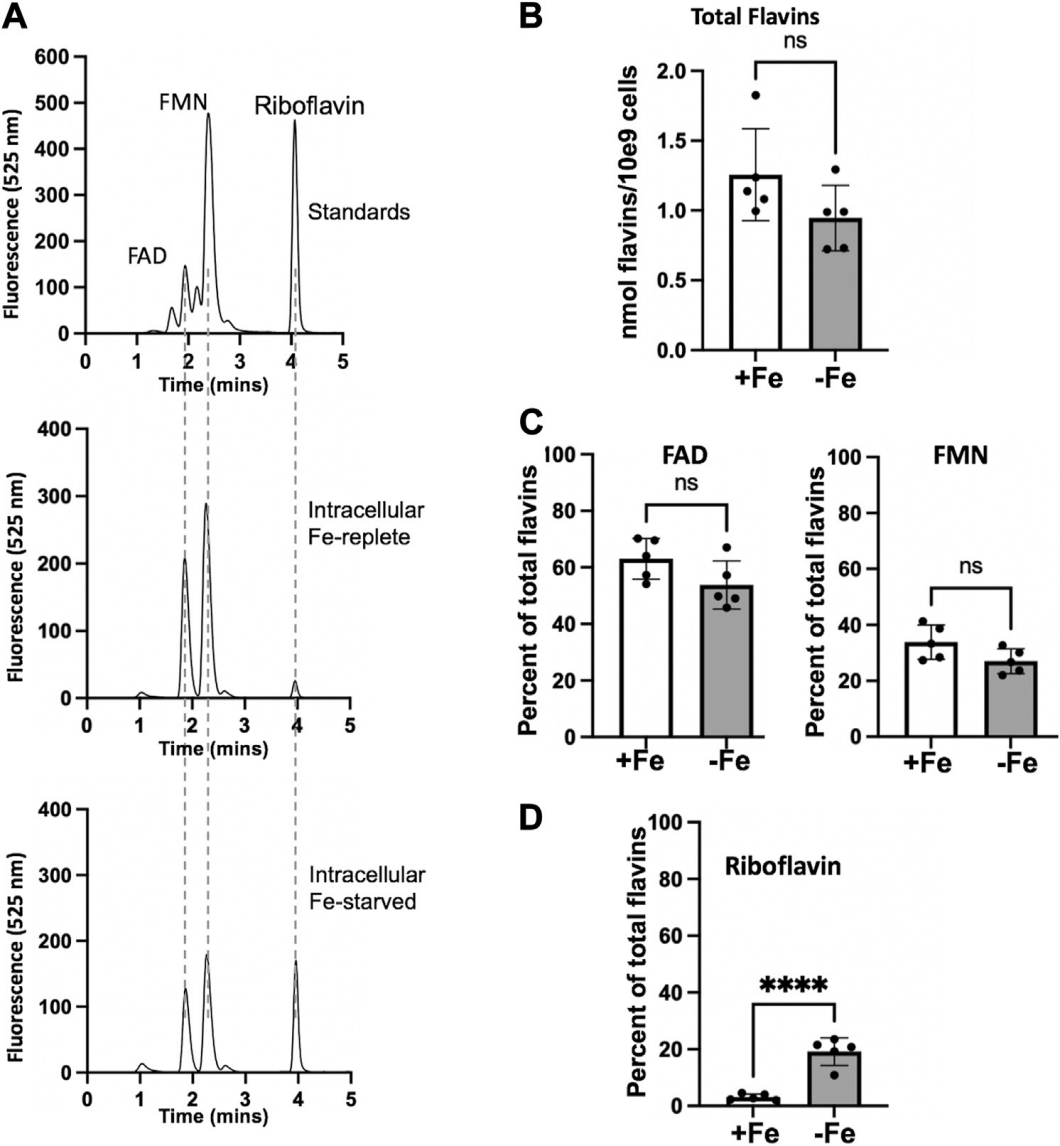
**The effects of Fe-starvation on intracellular flavins.***A*, metabolites were extracted from Fe-replete or Fe-starved cells as described in *Experimental Procedures* and subjected to HPLC analysis of flavins in comparison to standards as in Figure 1. *B*–*D*, Flavins were quantified as in Figure 1*E*. *B*, the sum of FAD, FMN and riboflavin peaks was used to derive total intracellular flavins, and (*C*, *D*) the percentage contribution was calculated for individual flavins in each culture. Each value represents an independent culture. Bars are mean and standard deviation, and significance determined by unpaired t-tests; ∗∗∗∗*p* < 0.0001; ns, *p* > 0.05. Strain utilized: SC5314.

Currently, nothing is known about the effects of Fe-starvation on intracellular pools of flavins in fungal cells. Do intracellular flavins also increase during Fe-starvation and if so, which ones? To address this, whole-cell flavins were extracted and subjected to HPLC and fluorescence analysis. As seen in Figure 2*A*, FAD and FMN are the predominant flavins in Fe-replete cells, as expected based on the role of these molecules as enzyme cofactors for redox chemistry (32). By comparison, riboflavin is barely detectable in Fe-replete cells (Fig. 2*A*). Upon quantification, we observe that in Fe-replete cells, the level of total flavins is similar to that reported for *Saccharomyces cerevisiae* (Fig. 2*B*) (29), as are the percent contributions of the individual flavins (Fig. 2, *C* and *D*) (29). FAD predominates in Fe-replete *C. albicans*, accounting for >50% of total intracellular flavins, with FMN representing the second most abundant flavin (Fig. 2*C*). Riboflavin constitutes only a few percentage of the total flavin pool in Fe-replete cells (Fig. 2*D*), similar to findings with Fe-replete *S. cerevisiae* (29).

When *C. albicans* is starved of Fe, there is a marked increase in intracellular riboflavin but not other flavins (Fig. 2*A*). When quantified, we observed a nearly 6-fold increase in riboflavin levels with Fe-starvation, to a point where riboflavin represents nearly 20% of total cellular flavins (Fig. 2*D*). With an increase in riboflavin, there is a trend toward a decrease in FAD and FMN under Fe-starvation (Fig. 2*C*). Overall, the main impact of Fe-starvation on flavins is an increase in riboflavin, both intracellularly and extracellularly, with marginal effects on lowering FAD and FMN.

### The role of Sef1 in Fe control of intracellular and extracellular flavins

**Figure 3:**
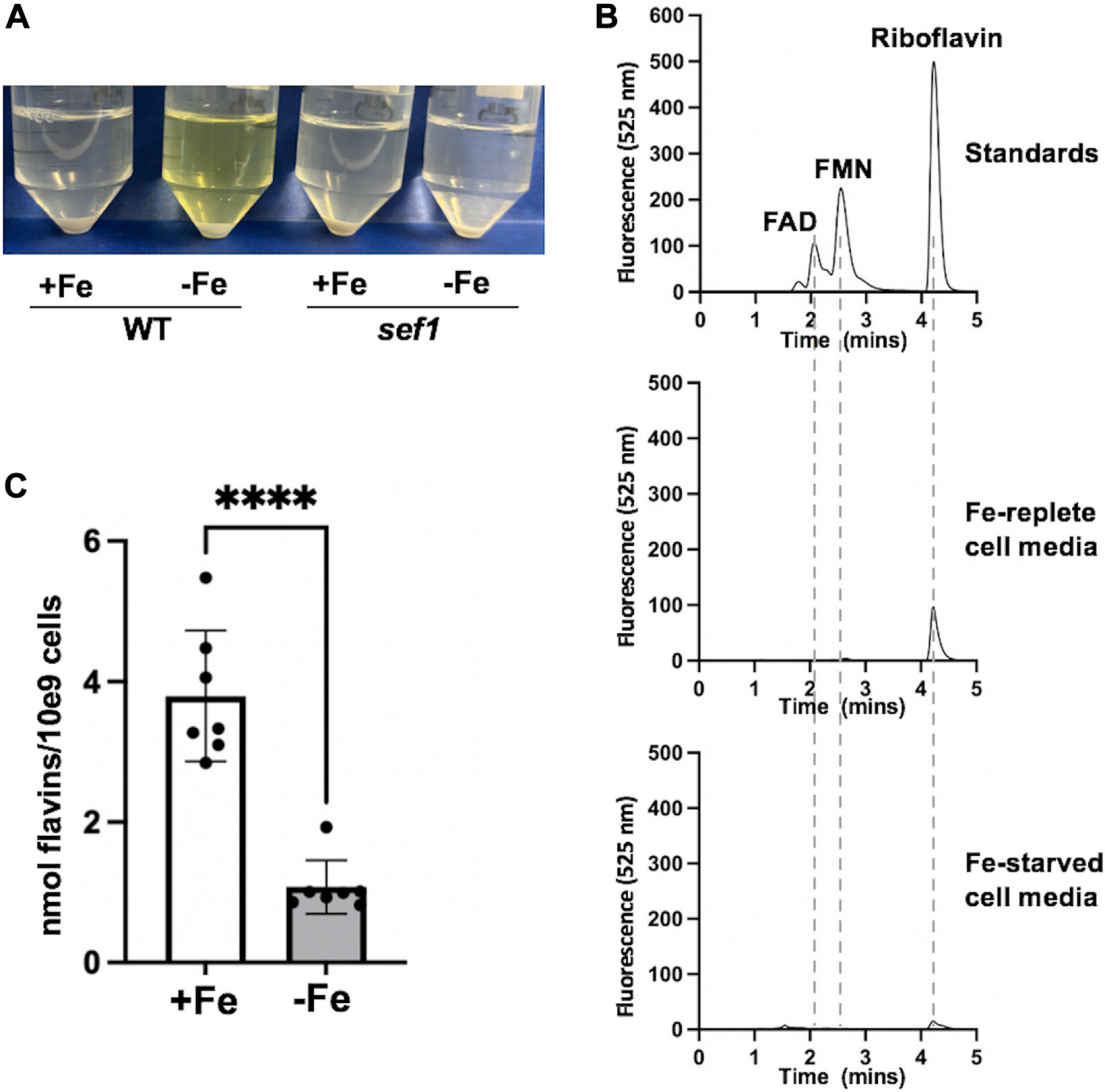
**The dependence on SEF1 for flavin secretion during Fe-starvation.***A*, picture of WT and *sef1Δ/Δ* cultures grown under Fe-starvation (−Fe) or Fe-replete (+Fe) conditions, showing the intense *yellow* color of secreted flavins from WT Fe-starved cells. *B*, HPLC chromatograms of flavin standards and the spent media of *sef1Δ/Δ* mutants were obtained as in Figure 1*A*. *C*, extracellular riboflavin was quantified as in Figure 1*E*. Each value represents an independent fungal culture. Bars are mean and standard deviation, and significance determined by unpaired *t* test; ∗∗∗∗*p* < 0.0001. Strains utilized: WT, SC5314 (*A*) or the *sef1Δ/Δ* derivative of SC5314 (“*sef1”* in *A*, and *B*, *C*).

The transcription factor Sef1 is known to be necessary for the secretion of flavins in *C. albicans* under Fe-starvation conditions (15, 16). However, any role of Sef1 under Fe-replete conditions or in controlling intracellular flavin pools has not been examined. We therefore investigated individual flavins in *sef1Δ/Δ* mutants. Although *sef1Δ/Δ* mutants are defective in inducing major Fe uptake genes during Fe starvation (14), they show no deficiency in total Fe accumulation (Fig. 1*A*), consistent with previous studies (14), and also no loss in Cu and Mn (Fig. 1, *B* and *C*). Cu and Mn levels are known to decline in *C. albicans* as cell density increases (33), and the higher Cu and Mn of Fe-starved *sef1Δ/Δ* cells as seen in Figure 1, *B* and *C* may reflect the lower cell density of these cultures (see Experimental Procedures). As seen in Figure 3*A*, Fe-depleted *sef1Δ/Δ* cultures fail to produce the characteristic yellow color of secreted flavins, consistent with previous studies that used metal chelators to induce Fe-starvation (15, 16). Upon HPLC analysis of the extracellular media, we observed low levels of secreted riboflavin in Fe-replete *sef1Δ/Δ* mutants (Fig. 3*B* middle). Upon quantification, this level of 3 to 4 nmol/10^9^ cells approximates that of Fe-replete WT cultures (Figs. 3*C* and 1*E*). Unexpectedly, we observed a striking repression of riboflavin secretion in Fe-starved *sef1Δ/Δ* mutants, and riboflavin levels secreted per cell number consistently decreased ≈3-fold (Fig. 3, *B* and *C*). These findings are in direct opposition to the strong upregulation of riboflavin secretion in Fe-starved WT cells (Figs. 3*A* and 1*H*).

**Figure 4:**
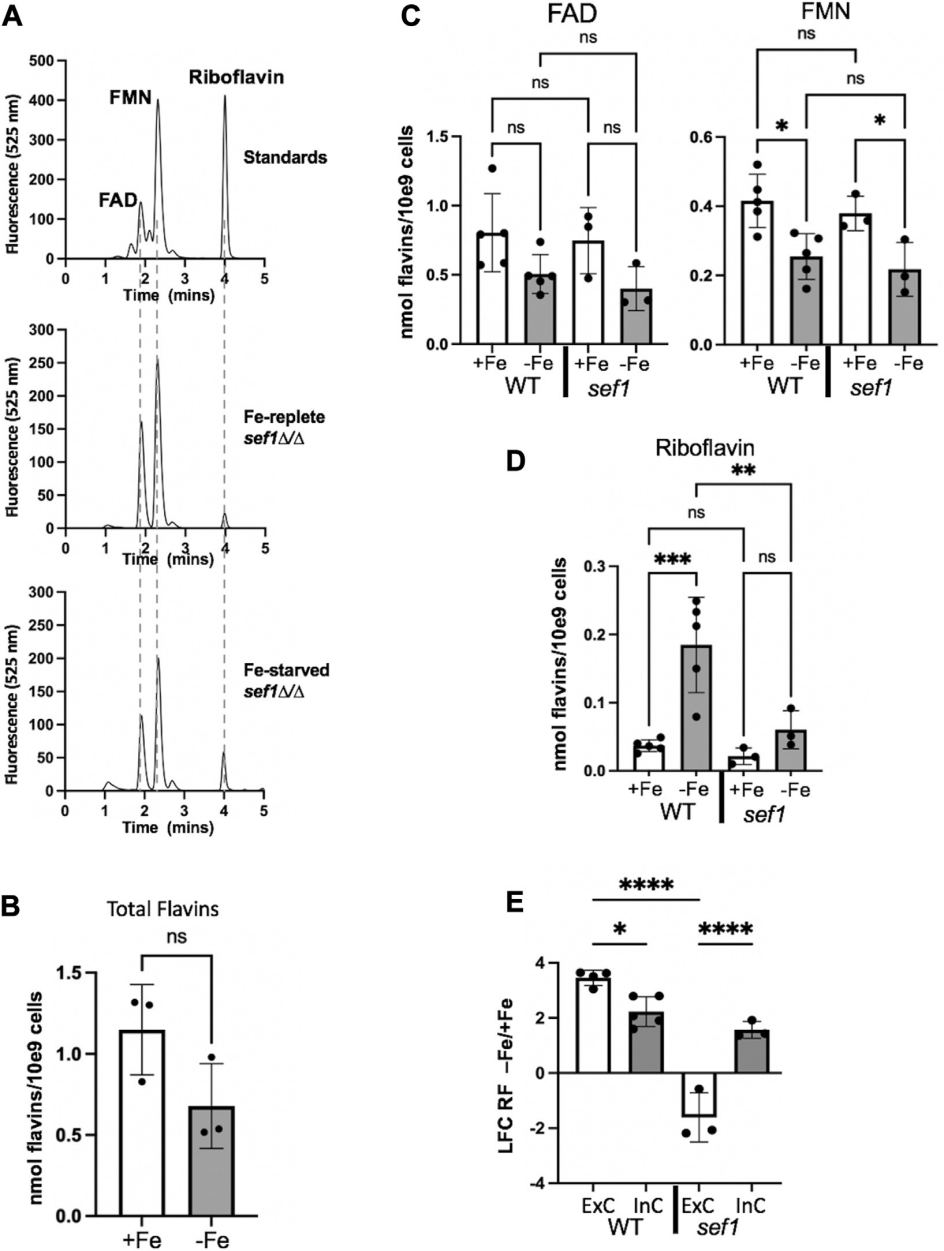
**Effects of *sef1Δ/Δ* mutations on intracellular flavins during Fe-starvation.***A*, HPLC chromatograms of flavin standards (*top*) and of intracellular flavins extracted from *sef1Δ/Δ* Fe-replete (*middle*) and Fe-starved (*bottom*) cells as obtained in Figure 2*A*. *B*, total flavins were quantified from three individual pairs of Fe-starved and Fe-replete *sef1Δ/Δ* cultures as in Figure 2*B*. *C* and *D*, quantification of individual flavins from WT *versus sef1Δ/Δ* cultures was carried out as in Experimental Procedures and Figure 1*E*. *E*, for each pair of Fe-replete and Fe-starved cultures grown in parallel, the log2 fold change (LFC) in riboflavin from Fe-replete vs Fe-starved cells was calculated for intracellular (“InC”) and extracellular (“E x C”) riboflavin. Results show opposing effects of Fe-starvation on intracellular *versus* extracellular riboflavin of *sef1Δ/Δ* mutants. Significance was determined by one-way Anova with *post hoc* Tukey test and also by an unpaired *t* test in the case of *sef1Δ/Δ* FMN (part *C*); ∗∗∗∗*p* < 0.0001; ∗∗∗*p* = 0.0003; ∗∗*p* = 0.0035; ∗*p* = 0.013 (WT FMN -Fe vs + Fe) or 0.0395 (*sef1* FMN -Fe vs + Fe) or 0.039 (part E); ns *p* > 0.05. Strains utilized: SC5314 (*C*–*E*) or the *sef1Δ/Δ* derivative (*A*–*E*).

We additionally examined intracellular pools of flavins in *sef1Δ/Δ* mutants. As in WT cells, FAD and FMN predominate in Fe-replete *sef1Δ/Δ* cells, with relatively minor contributions from riboflavin (Fig. 4*A*). The total level of intracellular flavins in Fe-replete and Fe-starved *sef1Δ/Δ* mutants (Fig. 4*B*) is very similar to that of WT cells (Fig. 2*B*), as are the levels of FAD and FMN, including trends towards a lowering of FAD and FMN with Fe-starvation (Fig. 4*C*). Furthermore, intracellular riboflavin levels in Fe-replete *sef1Δ/Δ* mutants were very comparable to that of Fe-replete WT cells (Fig. 4*D*). We expected that with Fe-starvation, *sef1Δ/Δ* cells would exhibit a lowering of intracellular riboflavin, to match the decreased extracellular riboflavin of these Fe-starved cells (Fig. 3*C*). However, if anything, intracellular riboflavin of *sef1Δ/Δ* mutants increased with Fe-starvation (Fig. 4, *A* and *D*). In examining log2 fold changes in riboflavin from Fe-starved *versus* Fe-replete *sef1Δ/Δ* cells, we observed equal but opposite effects with intracellular and extracellular riboflavin (Fig. 4*E*). The elevation in intracellular riboflavin of Fe-starved *sef1* cells was accompanied by a decrease in extracellular pools, a reversal of results with WT cells where the flavin rises both inside and outside the cell with Fe-starvation (Fig. 4*E*). These findings suggest that *sef1Δ/Δ* mutants may suppress riboflavin export during Fe-starvation, leading to an increase in intracellular, but losses in extracellular pools, of this flavin.

**Figure 5:**
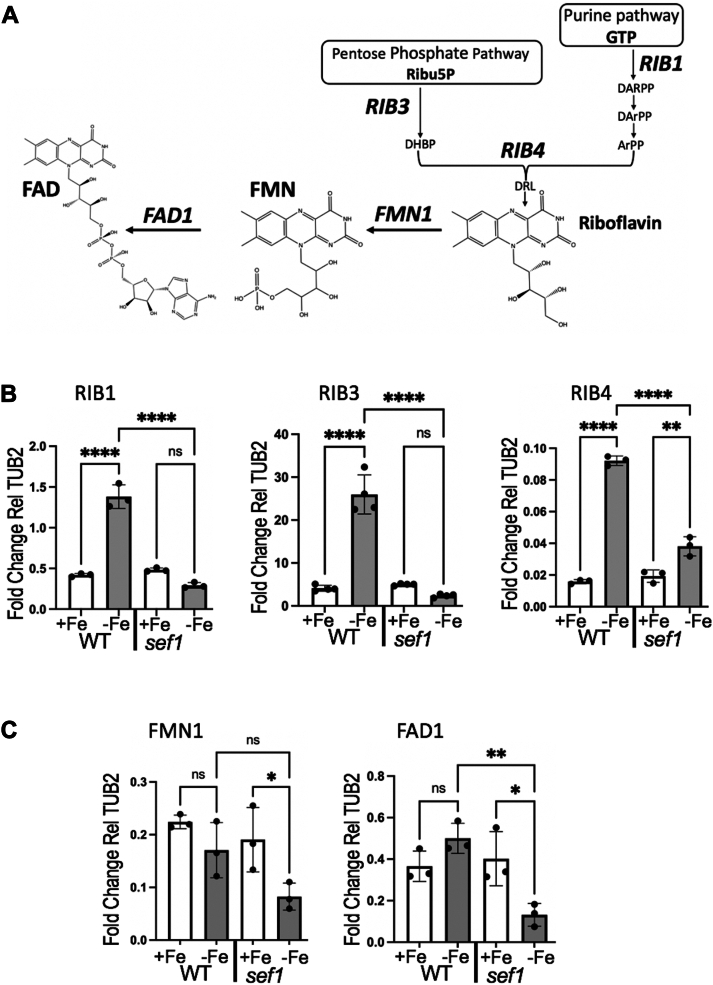
**Expression of riboflavin biosynthesis and flavin conversion genes.***A*, shown is the pathway for riboflavin biosynthesis from GTP and ribulose sugars, and the steps involving *C. albicans RIB1, RIB3* and *RIB4* genes. Riboflavin is converted to FMN and FAD by the Fmn1 flavin kinase and FAD synthase Fad1, respectively. See the main text for details. *B*, qRT-PCR analysis of the designated riboflavin biosynthesis and flavin conversion genes was carried out with the indicated strains grown in Fe-starvation or Fe-replete conditions. The results shown are compared to *TUB2* expression. Each value represents an independent fungal culture. Significance was determined by one-way Anova with *post hoc* Tukey test and also by an unpaired *t* test in the case of *sef1Δ/Δ FMN1*. ∗∗∗∗*p* < 0.0001; ∗∗*p* = 0.0015 (part *B*) and 0.0034 (part *C*); ∗*p* = 0.048 (part *C*, FMN1) and 0.012 (part *C*, FAD1); ns *p* > 0.05. Strains utilized: SC5314 and the *sef1Δ/Δ* derivative.

To better understand the role of Fe-starvation and Sef1 in controlling flavins, we examined the expression of flavin biosynthetic genes. Riboflavin biosynthesis requires one molecule of GTP and two molecules of ribulose 5-phosphate (Ribu5p) from the pentose phosphate pathway and involves several *RIB* encoded enzymes (Fig. 5*A*). In *C. albicans*, GTP is converted to 5-amino-6-ribitylamino-2,3-pyrimidinedione 5′-phosphate (Arpp) through a series of reactions starting with the GTP cyclohydrolase II Rib1 (28, 34). In a separate branch, Rib3 converts Ribu5p into 3,4-dihydroxy-2-butanone 4-phosphate (DHBP). The two pathways then converge in a reaction catalyzed by Rib4, leading to the production of 6,7-dimethyl-8-ribityllumazine (DRL) and then riboflavin (Fig. 5*A*). Rib1 has been characterized as the rate-limiting step (15), and both *RIB1* and *RIB4* are strongly induced by Fe-starvation in WT cells (Fig. 5*B*), similar to previous findings with Fe chelators (15, 16). *RIB3* is also strongly induced by Fe-starvation in WT cells (Fig. 5*B*). However, in the *sef1Δ*/Δ mutant, neither *RIB1* nor *RIB3* was induced with Fe-starvation and while *RIB4* expression increased ≈50%, this level was dwarfed by comparison to the 5- to 10-fold upregulation of *RIB4* in Fe-starved WT cells (Fig. 5*B*). We also examined expression of the flavin conversion genes, *FMN1* and *FAD1*. In WT cells, neither *FMN1* nor *FAD1* was significantly induced by Fe-starvation, and if anything, *FMN1* exhibited a trend towards down-regulation with Fe-starvation (Fig. 5*C*). Interestingly, in the *sef1Δ* mutant, both flavin conversion genes were significantly downregulated under Fe-starvation (Fig. 5*C*).

**Figure 7:**
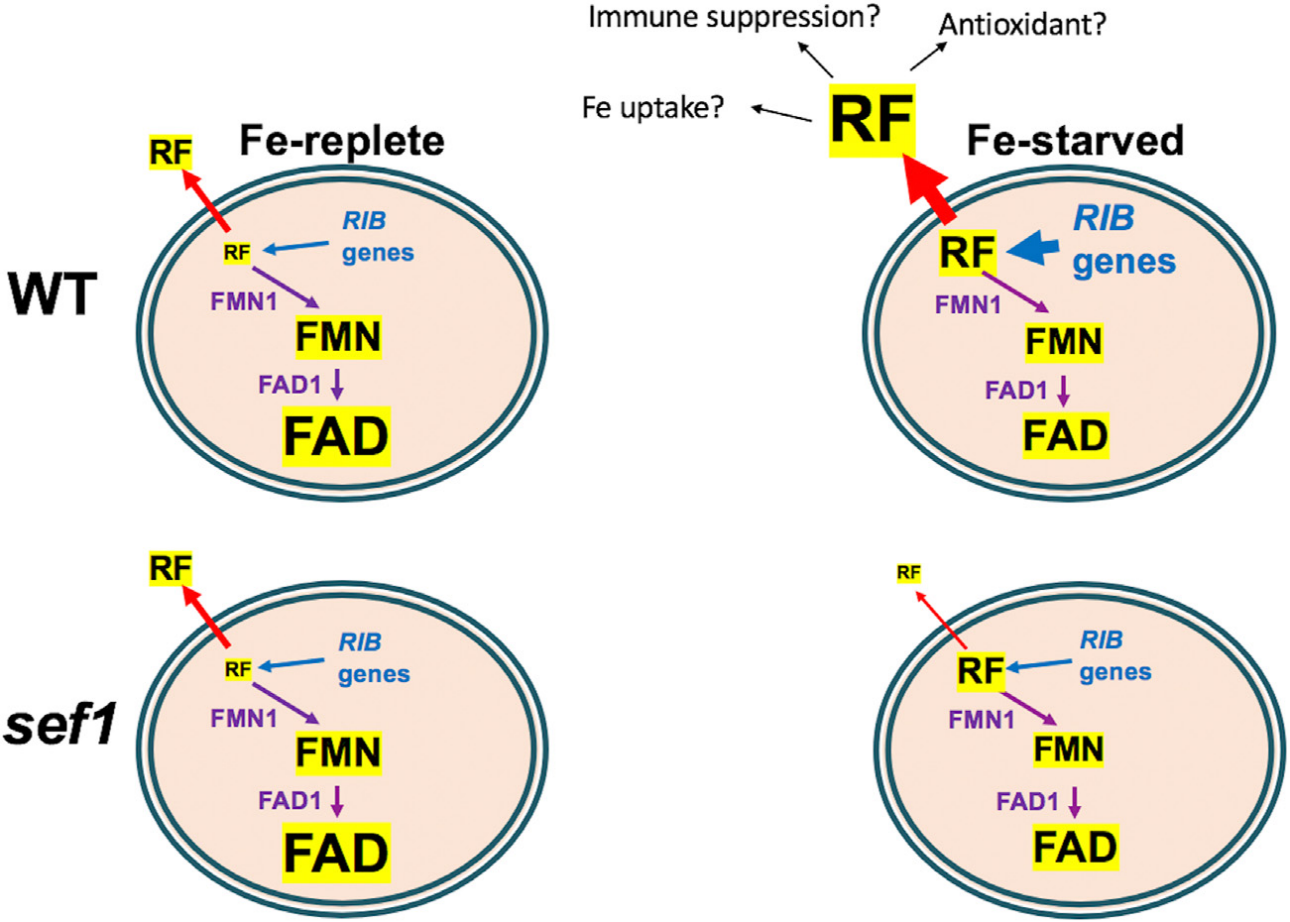
**Model for the crosstalk between Fe and flavins in pathogenic *C. albicans*.** Under Fe-replete conditions, FAD and FMN are the predominant intracellular flavins *of C. albicans*, with trace riboflavin (RF) and very little riboflavin is secreted. (Font size of FAD, FMN, and RF reflects relative abundance.) When Fe-starved, WT cells upregulate *RIB* genes for riboflavin synthesis (depicted in *blue*) but not flavin conversion genes (*purple*). Riboflavin levels rise inside the cell with Fe-starvation, but this is not accompanied by an increase in FMN and FAD. Rather, the excess riboflavin is exported from the cell in Fe-starved WT cells. Sef1 is required to induce riboflavin biosynthesis as well as riboflavin export, and mutants of *sef1Δ/Δ* show an increase in intracellular RF, but a repression in RF export during Fe-starvation. The riboflavin exporter regulated by Fe and Sef1 (*red arrow*) is still unknown. The cartoon also depicts possible roles for secreted riboflavin in Fe-starved *C. albicans*, including increased Fe uptake from serum as documented in our studies, and activities as an immune suppressant and antioxidant (see main text for details).

Overall, these changes at the mRNA level are consistent with findings of Figure 1, Figure 2, Figure 3, Figure 4 on flavin metabolites. Fe-starvation strongly induces genes for riboflavin biosynthesis in WT cells but not genes for flavin conversion (Fig. 5, *B* and *C*). As a result, intracellular riboflavin rises, but not FMN and FAD, and the elevated riboflavin in Fe-starved WT cells is exported (Figs. 1, 2 and see ahead cartoon of Fig. 7). By comparison, *sef1Δ/Δ* mutants do not exhibit the same strong upregulation in riboflavin biosynthesis genes and if anything, the genes for flavin conversion are downregulated during Fe-starvation (Fig. 5, *B* and *C*), consistent with losses in FMN and FAD (Fig. 4*C*, cartoon of Fig. 7). The major difference between WT and *sef1Δ/Δ* mutant cells lies in riboflavin export. While much of the riboflavin produced during Fe-starvation is exported from WT cells, riboflavin export appears repressed in Fe-starved *sef1Δ/Δ* mutants (Fig. 3*C*), with an accompanying increase in intracellular riboflavin (Fig. 4, *D* and *E*, cartoon of Fig. 7).

A remaining question regards the nature of the riboflavin exporter and its regulation by Fe and Sef1. The mechanism for riboflavin export from *C. albicans* and other flavogenic organisms is poorly understood. An importer for riboflavin, Rut1, was recently identified in *C. albicans* as a member of the monocarboxylic transporter family, but the exporter remains elusive (28). The only well-characterized riboflavin exporter is *BCRP*, an ABC transporter in human breast (35). A putative fungal homologue has been identified as Rfe1 (*C. albicans* orf19.3120) (36, 37), although by transcriptome analysis, this gene is expressed at negligible levels in *C. albicans* (14). Rfe1 is a member of a large family of 19 ABC transporters in *C. albicans* involved in drug resistance and export of other metabolites (38). If an ABC transporter is indeed responsible for exporting riboflavin, as shown in other organisms, we expect this transporter to be up-regulated by Fe-starvation in a Sef1-dependent manner. Using an available RNA-seq dataset from WT and *sef1Δ/Δ* cells grown under Fe-replete and Fe-starvation conditions (14), we examined the 19 ABC transporters from *C. albicans*. As seen in Figure S2*B*, none of these displays Sef1-dependent induction by Fe starvation, with the possible exception of *MDL2*, of unknown function. However, Mdl2 is a predicted half-ABC transporter (only one membrane-spanning domain) (38), and the degree of Fe-regulation is modest (<2-fold) (Fig. S2*B*). The Fe-control of riboflavin export may lie at the post-mRNA level, and identification of the transporter is worthy of future study.

### Riboflavin and Fe uptake

**Figure 6:**
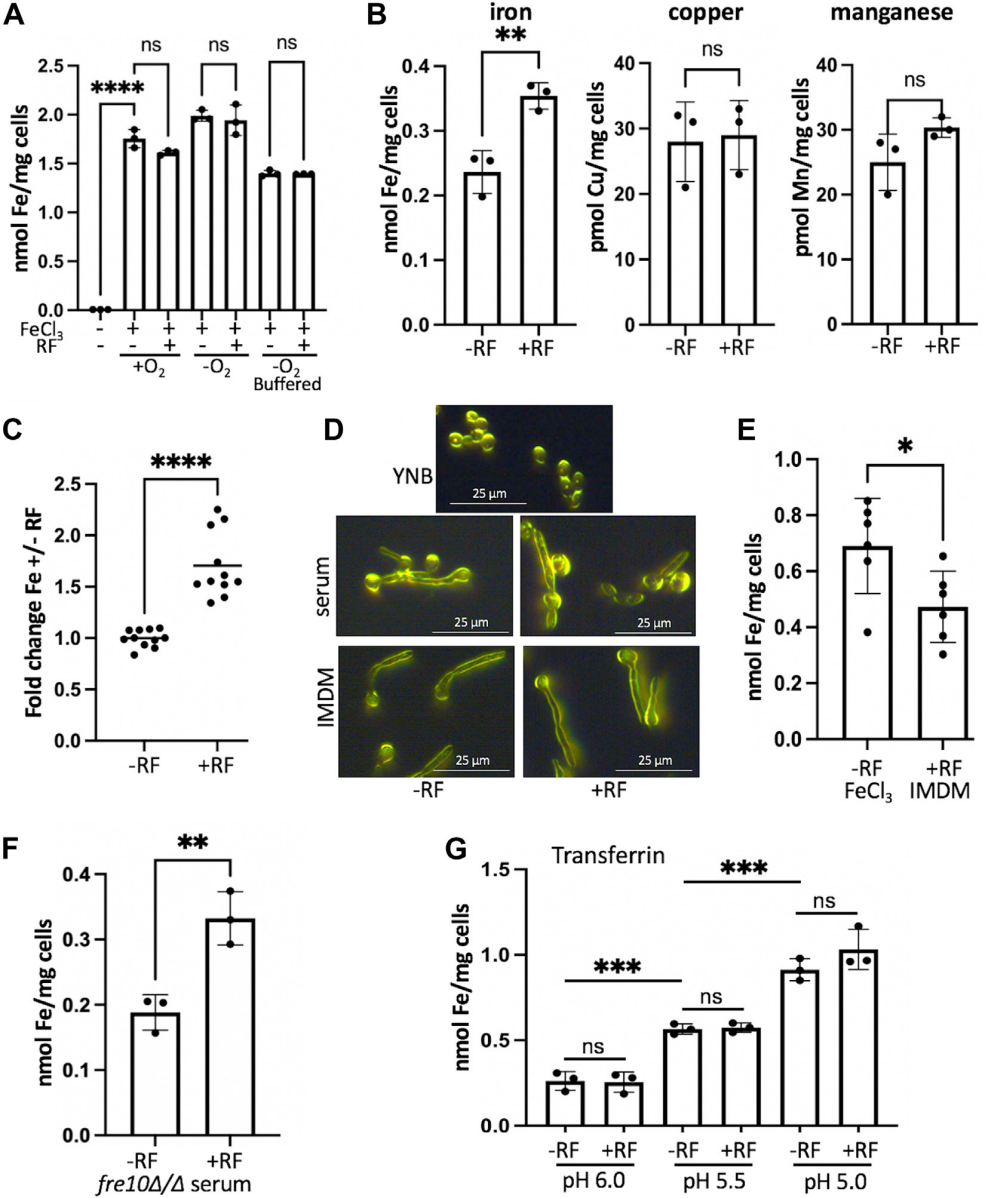
**Effects of riboflavin on Fe uptake in *C. albicans*.** The WT strain SC5314 (*A*–*E*, *G*) and *fre10Δ/Δ* mutant (*F*) were assayed for either Fe-accumulation (*A*–*C*, *E*–*G*) or for cell morphology by dark field microscopy (*D*) as described in Experimental Procedures. The Fe uptake studies used the following sources of Fe: (*A*) 35 μM FeCl_3_ added to a yeast nitrogen base (YNB) media under aerobic (+O_2_) or anaerobic (−O_2_) conditions in unbuffered media or media "buffered" to pH 5.8 as indicated; (*B*, *C*, *F*) 50% fetal calf serum in YNB media; (*E*) 35 μM FeCl_3_ added to IMDM; (*G*) 5 μM diferric-transferrin added to IMDM at the indicated pH. With the exception of (*A*), which was conducted at 30 °C, all experiments were carried out at 37 °C under anaerobic conditions. Accumulation of Fe (*A*–*C* and *E*–*G*) or of Mn and Cu (*B*) was monitored in cells cultured in the absence or presence of 10 μM riboflavin (+RF). Individual values represent independent fungal cultures. In (*C*), values were plotted relative to the no-riboflavin mean for each experimental trial. Significance was determined by one-way Anova with *post hoc* Tukey test (*A*) or unpaired t-tests (*B* and *C*, *E*–*G*). ∗∗∗∗*p* < 0.0001; ∗∗∗*p* = 0.0007 (part *G* -RF pH 6.0 vs pH 5.5) and 0.0002 (part *G* -RF pH 5.5 vs pH 5.0); ∗∗*p* = 0.0064 (part *B*) and 0.0084 (part *F*); ∗*p* = 0.031, ns *p* > 0.05. (*D*) Shown are images of cell morphology from the various Fe uptake studies. In experiments with 35 μM FeCl_3_ in YNB, cells are in yeast form. Serum induces the formation of short hyphae/germ tubes, as does IMDM. The addition of riboflavin (RF) does not alter overall cell morphology.

Why do cells secrete riboflavin during Fe-starvation? It has been hypothesized that flavins are secreted to reduce extracellular insoluble ferric Fe^3+^ to ferrous Fe^2+^ that can then be taken up by the cell (21, 37). However, no studies to date have shown riboflavin's assistance with Fe uptake. Instead, negative results have been reported with a lack of any effect of added riboflavin on Fe uptake in certain bacterial species (22, 25). Effects of flavins on fungal Fe uptake have not been previously documented, and we sought to understand whether riboflavin would increase Fe uptake in *C. albicans*. The genes for riboflavin biosynthesis in *C. albicans* are all essential (28), but we can suppress riboflavin secretion by culturing cells under Fe-replete conditions. In the experiments of Figure 6, cells were first cultured under low Fe conditions to induce genes for Fe import, then switched to Fe-replete conditions by supplementing Fe in the form of 35 μM FeCl_3_ or 50% fetal bovine serum (containing 25–70 μM Fe depending on the commercial lot) (39). We then tested whether riboflavin can enhance Fe acquisition from either FeCl_3_ or serum by supplementing 10 μM riboflavin, a level that typically accumulates in the media of Fe-starved *C. albicans* (Fig. S2*C*). Figure 6*A* shows Fe acquisition from FeCl_3_ involving the reductive Fe import pathway. Cells accumulated abundant Fe from FeCl_3_; however, there was no increase in Fe uptake with riboflavin (Fig. 6*A*). Riboflavin is largely oxidized in air, and any reduction of ferric Fe would require the reduced form of riboflavin (21). Yet even under anaerobic conditions, which should favor riboflavin reduction, there was no enhancement in Fe uptake from FeCl_3_ with riboflavin (Fig. 6*A*). *C. albicans* acidifies its extracellular environment, and cultures grown in unbuffered synthetic media are typically at very low pH ∼ 2.0 to 4.0. However, even in synthetic media buffered to a higher pH of 5.8, there is no enhancement of Fe uptake from FeCl_3_ by riboflavin (Fig. 6*A*).

Compared to FeCl_3_, animal serum is a more physiological source of Fe for *C. albicans.* Unexpectedly, the addition of riboflavin did enhance Fe acquisition from serum by roughly 50% (Fig. 6*B*). By comparison, there was no effect of riboflavin on the accumulation of redox-active Cu or Mn in the presence of serum (Fig. 6*B*). The effect of riboflavin on Fe uptake was reproducible with independent lots of commercial fetal bovine serum (Fig. 6*C*). The most striking results were obtained with cells at 37 °C under low oxygen (Fig. 6, *B* and *C*), although a limited degree of enhancement was also seen under other conditions, *e.g.*, 30 °C in air (Fig. S2*D*). *C. albicans* can exist in multiple morphological states from rounded yeast-form cells to filamentous hyphae and pseudo hyphae. In the synthetic yeast nitrogen base (YNB) media as used in the FeCl_3_ uptake studies of Figure 6*A*, cells are in yeast form (Fig. 6*D* top). However, serum induces hyphal formation (40, 41), and in the serum Fe uptake studies of Figure 6, *B* and *C*, cells exhibit germ tube/short hyphae morphology (Fig. 6*D*, middle). To test whether hyphal formation accounts for the enhancement effects of riboflavin, we induced morphogenesis independent of serum using Iscove's Modified Dulbecco's Medium (IMDM), a synthetic medium with very low (≈50 nM) Fe that induces hyphae through high amino acids (42, 43). The cells produced short hyphae in IMDM supplemented with FeCl_3_ (Fig. 6*D* bottom), but riboflavin did not enhance hyphal cell uptake of Fe from FeCl_3_ (Fig. 6*E*). These studies demonstrate that hyphal formation by itself is not the driving force for riboflavin enhancement of Fe uptake. Rather, riboflavin is acting on a specific Fe source(s) in animal serum, presumably by reducing this Fe^3+^ source to a more bioavailable Fe^2+^ form.

Transferrin is one of the major Fe^3+^ sources in serum, and *C. albicans* can extract Fe from transferrin (9). We therefore tested whether the riboflavin effects on Fe uptake involved serum transferrin. The transferrin uptake pathway in *C. albicans* requires the ferric reductase Fre10 (9); however, *fre10Δ/Δ* mutations do not prevent the riboflavin enhancement of Fe uptake in serum (Fig. 6*F*). We also tested whether riboflavin could facilitate Fe acquisition from purified transferrin by supplementing diferric transferrin as a sole Fe source to hyphal cells grown in IMDM. Since Fe release from transferrin is pH dependent (44), the studies were conducted at pH ranges approaching that of cultures in serum (pH 5.8) as well as a lower pH value. Although the hyphal cells accumulated abundant Fe from transferrin, and Fe uptake increased with a lowering in pH, there was no effect of supplemental riboflavin on Fe uptake from transferrin (Fig. 6*G*). Together, these studies imply that transferrin is not the component of serum that serves as the target of riboflavin, and other Fe sources must be involved. There are numerous Fe^3+^ containing molecules in serum, including hemoglobin/heme, ferritin, and a broad class of factors collectively known as NTBI or non-transferrin-bound Fe that loosely bind Fe (45, 46). One or more of these Fe^3+^ sources may serve as the target of redox-active riboflavin for enhancing Fe acquisition from serum.

It is important to note that the effect of riboflavin on increasing Fe accumulation *in vitro* is relatively modest (≈50%) (Fig. 6, *B* and *C*). Even without added riboflavin, cells accumulate much Fe from serum (Fig. 6*B*). Riboflavin is not the only redox molecule that may reduce serum Fe^3+^ to Fe^2+^ for metal uptake, and *C. albicans* expresses a large family of FRE ferric reductase enzymes (47). It is currently unknown what contribution riboflavin would have on Fe accumulation inside the animal host, and we cannot exclude the possibility that *in vivo*, secreted riboflavin has an alternative purpose. *C. albicans* is an opportunistic fungal pathogen, and riboflavin is predicted to be secreted specifically in the infectious state, when the organism is subjected to host-imposed Fe-starvation as seen in the murine model of disseminated candidiasis (4, 5, 6, 10). There are a number of ways riboflavin might promote fungal fitness and pathogenesis. In addition to enhancing Fe uptake as described here, riboflavin could dampen the immune response to *C. albicans* invasion, as has been recently shown for certain viral or bacterial infections (48, 49). Riboflavin can also act as an antioxidant (50) and may help protect against the oxidative burst of host immunity (Fig. 7). With *C. albicans* and other flavogenic organisms, riboflavin is not just a precursor to FAD and FMN but has the capacity to modify the extracellular environment. These studies underscore the importance of studies evaluating the riboflavin biosynthesis pathway as a candidate target for future anti-fungal therapies (15, 28).

## Experimental procedures

### Strains and growth media

Unless noted otherwise, all studies involved the clinical isolate SC5314 and *sef1Δ/Δ* derivative provided by Joachim Morschhaeuser (16). The *fre10Δ/Δ* mutant was a kind gift of Andrew Dancis (9). Strains were maintained on YPD (1% yeast extract, 2% peptone, 2% dextrose) media, and all experiments used a synthetic media prepared as described (30) using yeast nitrogen base (YNB) lacking Fe and Cu (Sunrise Science 1526–250) and supplemented with 1.0 μM CuSO_4_. This YNB media as-is creates Fe-starvation conditions, and Fe-replete conditions were achieved by supplementing the YNB media with 5 μM FeCl_3_. All studies on flavin and mRNA analyses involved 10 ml *C albicans* cultures inoculated at an OD_600_ of 0.05 and grown for 24 h at 30 °C with shaking at 220 rpm. Under these conditions, cells reached stationary phase with a final OD_600_ of ≈10, with the exception of Fe-starved *sef1Δ/Δ* mutants that plateaued at an OD_600_ of ≈5. All measurements of flavins and metals described below were normalized for cell number.

### Flavin extraction and analysis by fluorescence, HPLC, and mass spectrometry

Cells for flavin metabolite extraction were grown in batches of five 10 ml cultures as described above. Following 24 h growth, cultures were chilled on ice and 75 OD_600_ cell units were subjected to centrifugation at 12*g* for 10 min at 4 °C. Cell pellets and spent media were stored in the dark at −80 °C until further processing. Extraction of metabolites from cell pellets used protocols adapted from Sasidharan *et al.* (51) for extracting whole-cell flavins. In brief, thawed cell pellets were washed twice with sterile Milli-Q water at 4 °C, and then resuspended in an extraction solution containing 150 μl chloroform, 225 μl of a 2:1 methanol: water mix, and roughly 100 μl of acid-washed glass beads. Cells were lysed at 4 °C by BeadBlaster 24 (Benchmark) at 4260 rpm with the following repeated 4 times: 3 cycles of 15 s on, 30 s rest, with 2-min breaks in between each cycle. Following centrifugation at 4 °C for 10 min, the aqueous layer was removed and combined from 5 cultures. Samples were concentrated to dryness overnight on a Thomas Savant ISS110 SpeedVac System on low drying rate settings. For analysis of extracellular flavins, 1 to 2.5 mls of spent media were concentrated to dryness as described above, except in the case of preparative experiments for mass spectrometry, where 5 mls of spent media was used. Dehydrated samples were stored at −80 °C in the dark.

All HPLC analyses were performed on an Agilent 1260 infinity II HPLC with a fluorescence detector using protocols adapted from Gliszczyńska *et al.* (29). The dehydrated spent media or whole-cell metabolite samples were rehydrated in 250 μl of a 70:30 mix of 0.05 M ammonium acetate (HPLC Grade, Fisher Scientific A639–500) and methanol (Fisher Chemical HPLC Grade A452–4), vortexed, and filtered using 0.22 μM cellulose acetate Spin-X centrifuge tube filters (Costar 8160). 100 μl of the sample was injected into an Agilent Poroshell C-18 column pre-equilibrated in 70:30 ammonium acetate: methanol, then fractionated with a flow speed of 1 ml/min in a linear gradient to a 30:70 ratio of ammonium acetate: methanol by 5 min, which was altered to 5:95 ammonium acetate: methanol from 6.6 to 9.6 min, then returned to a 70:30 ammonium acetate: methanol from 10.6 to 15.6 min. Flavins were detected by fluorescence with excitation at 450 nm and emission at 525 nm.

Each sample run on HPLC was accompanied by analysis of flavin standards in tandem. 20 μM standards of riboflavin, FAD, and FMN (Acros Organics 132350250, MP Biomedicals 0215112780, Sigma-Aldrich F2253, respectively) were prepared in the aforementioned 70:30 mix of ammonium acetate: methanol. Individual flavins as well as a mix containing all three were filtered, and 100 μl was subjected to HPLC analysis as described above.

Quantification of individual extracellular or intracellular flavins was accomplished by calculating the area under the curve (AUC) using Agilent ICL software and by comparison to the emission spectra AUC for 2 nmol riboflavin, FAD, and FMN standards run on HPLC in tandem. AUC was in the linear range for all concentrations measured.

For mass spectrometry analysis, the HPLC fractions corresponding to the sole fluorescent peak of media from Fe-starved cells were collected, concentrated to dryness as described above, and resuspended in 1 mL 50:50 methanol: water (Sigma-Aldrich Water for HPLC 270733-4L). As control, a blank was created from HPLC fractions at 1 to 2 and 8 to 9 min, lacking any fluorescence. Samples were subjected to electrospray ionization (ESI) mass spectrometry using a Waters Xevo G2-S Q-TOF mass spectrometer at the Johns Hopkins University Mass Spectrometry facility. The sample was infused using a syringe pump (10 μl/min). The instrument was run in the negative mode. Data was acquired for several minutes and then averaged and processed using Waters MassLynx V4.2 software. Leucine enkephalin peptide was also sprayed into the instrument to serve as a lock mass to correct for any mass drift.

In quantifying total extracellular flavins without HPLC, cells were removed by centrifugation from 48-h Fe-starved or Fe-replete cultures, and 300 μl of spent media was analyzed for fluorescence by excitation at 450 nm and emission at 525 nm on a 96-well Thermo Scientific Nunc MicroWell Nunclon Delta treated plate (ThermoScientific 137,101) using a Biotek Synergy HT plate reader. Concentration in terms of μM was determined by comparison to a riboflavin standard curve.

### mRNA analysis by qRT-PCR

For qRT-PCR, 10 OD_600_ cell units of *C. albicans* cells grown as described above were harvested, washed twice in water treated with DEPC, and subjected to acid phenol extraction of mRNA according to published methods (30). Samples were treated with DNAase using the RapidOut DNA Removal Kit (ThermoFisher Scientific K2981), and 1 μg RNA used for cDNA synthesis by the Maxima H Minus First Strand cDNA synthesis Kit (ThermoFisher Scientific K1652) per manufacturers recommendations. qRT-PCR was accomplished using the PowerUp SYBR Green Master Mix (Thermo Fisher Scientific A25742) to generate ≈200 bp amplicons from primer sequences listed in Table 1 (15, 52). *C. albicans TUB2* was used as a housekeeping gene.

**Table 1:** Gene primers used for qRT-PCR

Gene name	Primer direction	Sequence
TUB2	Forward	GAG TTG GTG ATC AAT TCA GTG CTA T
	Reverse	ATG GCG GCA TCT TCT AAT GGG ATT T
RIB1	Forward	GTG CCG ATA CAG TGG AAG C
	Reverse	CCC AAA TCA ACC AAG ATA GCC
RIB3	Forward	TAC CGT TAA GAG CCG TTC CA
	Reverse	CAA ATC ACC CCA GCA GGT T
RIB4	Forward	GGC ATT CCT GTT ATT TTT GGT G
	Reverse	TGT GCA TTT TGC CTT CAA TC
FMN1	Forward	TCT GAA CTA GGA ATC CCC ACA
	Reverse	TTC GTC ACA TTG GGC TGT TA
FAD1	Forward	CCG AAT GGC AAA TAC CAA CCA
	Reverse	ACT CTA CCC AAC CGC TCA TT

### Metal accumulation studies

For studies on the role of riboflavin in Fe uptake, cells were first grown in Fe-deplete media for 18 h to a final OD_600_ of 2 to 4. Cells were harvested and washed to remove extracellular riboflavin, and 2.0 OD_600_ units of cells were resuspended in 10 mL of either Fe-deplete YNB media containing 35 μM FeCl_3_ (unbuffered or buffered to pH 5.8 with Tris-HCl) or 50% fetal bovine serum (Sigma-Aldrich lot F6178 or F4135) or 10 mL IMDM (Gibco 21,056,023). Where indicated, cells were supplemented with 35 μM FeCl_3_ or 5 μM transferrin (Sigma-Aldrich T0665) as Fe sources, and in the case of transferrin experiments, the pH of IMDM was adjusted to 5.0, 5.5, or 6.0. Incubation proceeded at 3.5 h by shaking at 200 rpm at 30 °C or 37 °C, in air or under anaerobic conditions achieved by BD GasPak systems (Fisher Scientific B260680). Cells were harvested, washed in 10 mM Tris, 1 mM EDTA, pH 8.0, and twice in Milli-Q water, and pellets were weighed. Samples were resuspended in 600 μl 20% Optima grade nitric acid (Fisher Scientific A467–500) and digested overnight at 90 °C. Samples were diluted to 3% nitric acid, and Fe was quantified using the colorimetric bathophenanthroline disulfonate (BPS) assay as described (53). In the case of serum studies with riboflavin, samples were also analyzed for Mn and Cu by atomic absorption spectrometry (AAS) using an AAnalyst600 graphite furnace spectrometer (PerkinElmer).

Metals were additionally measured in cell samples prepared for flavin analyses. A total of 50 OD_600_ units of cells grown under Fe-replete or Fe-starvation conditions were harvested, washed, and digested in 1.2 mls 20% nitric acid as described above. Aliquots were diluted to 2 or 3% nitric acid for analysis of Mn and Cu by AAS or for Fe analysis by the BPS assay, respectively. The very low levels of Fe in Fe-starved cultures were also quantified by quantitative inductively coupled plasma mass spectrometry (ICP-MS) on an Agilent 8900 triple quadrupole instrument (Mass Spectrometry Center, University of Maryland, School of Pharmacy). For ICP-MS, samples digested in 20% nitric acid were diluted to 6% nitric acid. Fe measurements by ICP-MS and the colorimetric BPS assay yield identical results (14).

### Cell microscopy and statistical analysis

Fungal cell morphology was analyzed by dark field microscopy at 40X magnification on a Nikon Infinity 1 microscope, using 1.5 μm per 10 pixels for size scale.

All statistical analysis was carried out using GraphPad Prism 9. The student's *t* test was used for pairwise comparisons, and all other analyses involved ANOVA with *post hoc* Tukey test.

## Data availability

All data are contained within the manuscript and supporting information.

## Supporting information

This article contains supporting information (14).

## Conflict of interest

The authors declare that they have no conflicts of interest with the contents of this article.
