# Supplementary material for: Crosstalk between iron and flavins in the opportunistic fungal pathogen Candida albicans
Source: J Biol Chem. 2025 Jun 19;301(7):110396. doi: 10.1016/j.jbc.2025.110396 (PMC12275197; doi:10.1016/j.jbc.2025.110396)
Supplement: Supporting information [file mmc1.pdf]

Supporting information for

Crosstalk between iron and flavins in the opportunistic fungal pathogen *Candida albicans*

Marika S. David[1], Zhengkai Zhu[1], Maranda R. McDonald[1], Mohsen Badiee[1], I. Phillip Mortimer[2],

Anthony K. L. Leung[1] and Valeria C. Culotta[1]

Contents:

Figure S1: ESI mass spectrometry analysis of flavin secreted by *C. albicans*
Figure S2: Riboflavin export and the effect of riboflavin on Fe uptake from serum at 30°C in air

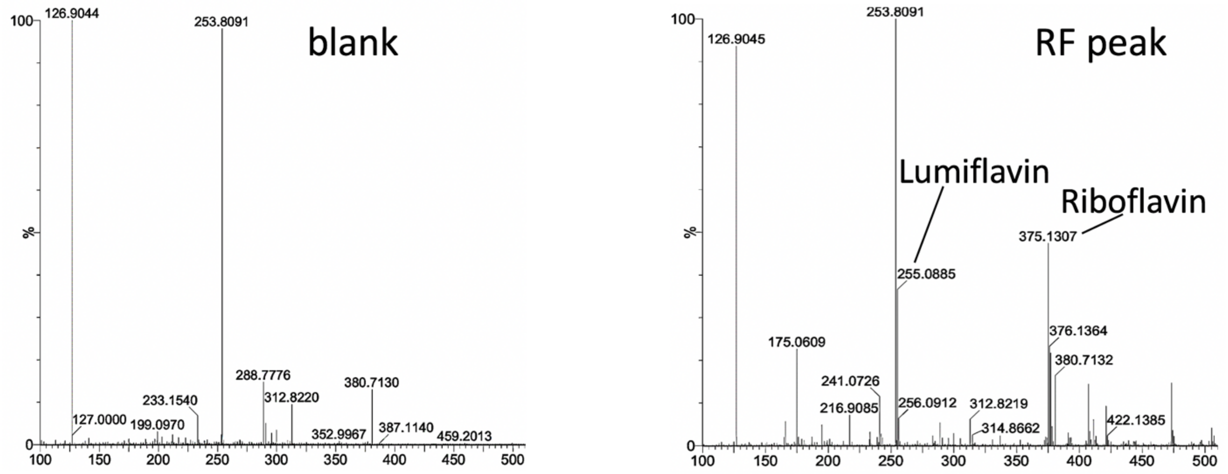

Figure S1: ESI mass spectrometry analysis of flavin secreted by *C. albicans*
Shown is the ESI mass spectrometry chromatogram of the HPLC fluorescent peak of Fe-starved WT cells (right) compared to a blank control (left) processed as described in *Experimental Procedures.* Riboflavin is known to undergo autolysis producing lumiflavin. The peaks at 253.8 m/z and 126.9 m/z are also in blank controls and are residual ions from CsI calibration solution.

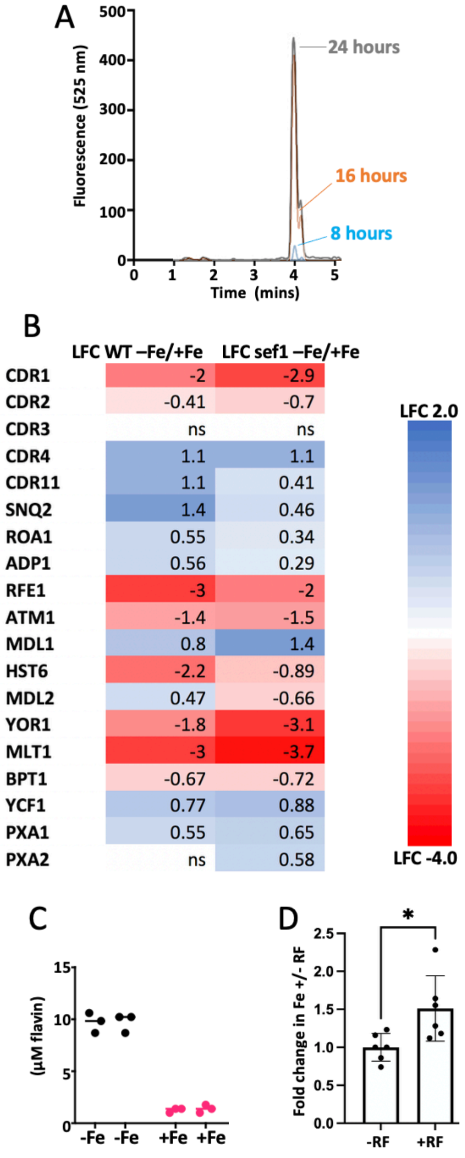

Figure S2: Riboflavin export and the effect of riboflavin on Fe uptake from serum at 30°C in air.

(A) Shown are superimposed HPLC chromatograms of extracellular riboflavin from Fe-starved cultures at 8, 16 and 24 hours, obtained as in Fig. 1A. (B) Heat map analysis of the family of MDR ABC transporters in *C. albicans.* Values indicate log2 fold change (LFC) in gene expression from Fe-starved versus Fe-replete (-Fe/+Fe) WT and *sef1Δ/Δ* cells as determined from a published RNA-seq data set (1). (C) Total flavins in µM levels were quantified from the unfractionated spent growth media of Fe-starved (black) and Fe-replete (pink) cultures as described in *Experimental Procedures.* Results are representative of two independent cultures with triplicate readings from each. (D) The effect of 10 µM riboflavin on *C. albicans* uptake of Fe from serum was carried out as in Fig. 6C, except the incubation with serum proceeded at 30°C in air.

1. Garg, R., Zhu, Z., Hernandez Francisco, G., Wang, Y., David Marika, S., Bruno Vincent, M., and Culotta Valeria, C. (2025) A response to iron involving carbon metabolism in the opportunistic fungal pathogen Candida albicans. *mSphere* **10**, e00040-00025
